# Liposomes to Cubosomes: The Evolution of Lipidic Nanocarriers and Their Cutting-Edge Biomedical Applications

**DOI:** 10.1021/acsabm.4c00153

**Published:** 2024-04-13

**Authors:** Nishtha Attri, Swarnali Das, Jhimli Banerjee, Shazana H. Shamsuddin, Sandeep Kumar Dash, Arindam Pramanik

**Affiliations:** † Amity Institute of Biotechnology, Amity University, Noida 201301, India; ‡ Department of Physiology, University of Gour Banga, Malda 732103, West Bengal, India; § Department of Pathology, School of Medical Sciences, Health Campus, Universiti Sains Malaysia, Kubang Kerian 16150, Kelantan, Malaysia; ∥ School of Medicine, University of Leeds, Leeds LS53RL, United Kingdom

**Keywords:** cancer therapeutic, cubosomes, lipidic nanoparticles, liposomes, solid lipid nanoparticles

## Abstract

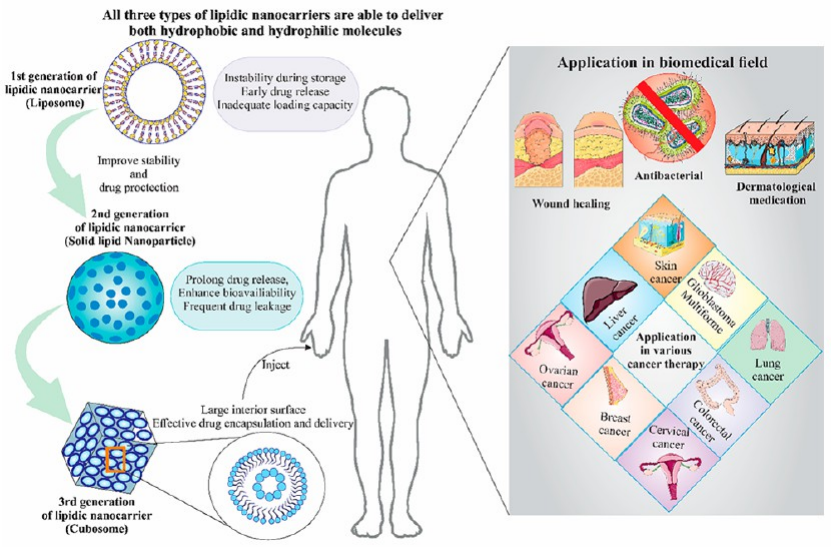

Lipidic nanoparticles have undergone extensive research toward the exploration of their diverse therapeutic applications. Although several liposomal formulations are in the clinic (e.g., DOXIL) for cancer therapy, there are many challenges associated with traditional liposomes. To address these issues, modifications in liposomal structure and further functionalization are desirable, leading to the emergence of solid lipid nanoparticles and the more recent liquid lipid nanoparticles. In this context, “cubosomes”, third-generation lipidic nanocarriers, have attracted significant attention due to their numerous advantages, including their porous structure, structural adaptability, high encapsulation efficiency resulting from their extensive internal surface area, enhanced stability, and biocompatibility. Cubosomes offer the potential for both enhanced cellular uptake and controlled release of encapsulated payloads. Beyond cancer therapy, cubosomes have demonstrated effectiveness in wound healing, antibacterial treatments, and various dermatological applications. In this review, the authors provide an overview of the evolution of lipidic nanocarriers, spanning from conventional liposomes to solid lipid nanoparticles, with a special emphasis on the development and application of cubosomes. Additionally, it delves into recent applications and preclinical trials associated with cubosome formulations, which could be of significant interest to readers from backgrounds in nanomedicine and clinicians.

## 1 Introduction

Lipid-based nanoparticles have drawn a lot of interest among different nanocarriers because of their biocompatibility, adaptability, and capacity to encapsulate hydrophobic medicines.^1^ Among lipidic nanocarriers, liposomes, solid lipid nanoparticles (SLNs), and liquid lipid nanoparticles (LLNs) have all been widely investigated.^2−4^ Because they are spherical vesicles made of phospholipid bilayers, liposomes could be utilized for the delivery of both hydrophilic and hydrophobic molecules.^5^ Due to the increased permeability and retention impact, their nanoscale size enables passive targeting and accumulation in tumor tissues.^6^ Several liposomal drug formulations, including those for doxorubicin (Dox), paclitaxel, and vincristine have been extensively studied and some have reached the clinic.^5^ Unfortunately, liposomes do have several limitations despite their advantages, including instability during storage, early drug release, and inefficient drug-loading capacity.^7^ Due to these limitations, several modifications of liposomes and new generations of lipidic nanocarriers have been developed. Solid lipid nanoparticles (SLNs), which are a second-generation lipidic nanocarrier, do address some of the issues of liposomes.^8^ SLNs have improved stability and drug protection because they have a solid lipid core that can be stabilized with surfactants. The solid matrix prolongs the release of the drug, enhances drug bioavailability, and at the same time stops drug leakage. Because these provide continuous and slow drug release, SLNs have demonstrated considerable promise in the treatment of cancer.^9^ Additionally, by conjugating ligands or antibodies that are specific to tumor markers, SLNs can be surface-functionalized to enable active targeting, further increasing their tumor accumulation and therapeutic effectiveness.^10^ The third generation of lipidic nanostructures are known as cubosomes which form lipid bilayers arranged into a bicontinuous cubic lattice.^11^ These special lipidic nanocarriers outperform conventional lipid-based nanoparticles in several ways, primarily because of their large interior surface area, which permits effective drug encapsulation and delivery.^12^ Cubosomes’ cubic form and their unique internal structure enable them to concurrently host both hydrophilic and hydrophobic therapeutic molecules.^13^ Their therapeutic potential is considerably increased by this aspect, which also makes it possible to develop personalized treatment plans for various cancers. Cubosomes are excellent candidates for combination therapy due to their capacity to carry a wide range of therapeutics, including chemotherapeutics, siRNA, and photosensitizers.^14−16^

The switch from liposomes to cubosomes has enhanced the potential for delivering genes in cancer therapy. RNA interference (RNAi) and gene editing are two nucleic acid–based mechanisms that have garnered popularity as possible cancer treatments.^17^ Nucleic acids may be effectively encapsulated and preserved by cubosomes, ensuring efficient transport to target cells. Additionally, the transport of both genes and drugs through a single cubosome carrier has synergistic therapeutic benefits that improve the overall success of the treatment.^18^ This review focuses on the biomedical research transition from liposomes, the first generation of lipidic nanocarriers, toward the development of the second generation of solid lipid nanoparticles, and finally to the most recent advances in the new generation of cubosome nanocarriers.

## 2 Liposomes As a Therapeutic Carrier

Liposomes, which are composed of one or more concentric lipid bilayers enclosing an aqueous compartment were first discovered in the 1960s.^19^ Liposomes are made of phospholipids, which are amphiphilic molecules, i.e., they contain a hydrophilic head and two polar hydrophobic chains. Out of many applications of liposomes, an important aspect of liposomes is its potential as a drug delivery system. This is solely dependent on the physicochemical characteristics of their membranes, the makeup of their constituent parts, as well as their size, surface charge, and lipid structure.^20^ Phospholipids have a great propensity to form membranes when dispersed in aqueous solutions because of their amphipathic character.^21^ Liposomes contain a hydrophilic cavity and a hydrophobic bilayer. They can therefore contain a variety of hydrophilic and hydrophobic molecules, including pharmaceutical drugs, imaging, and diagnostic agents.^22^ Although various nanoparticular structured systems have been created yet liposomes have outperformed them due to several undeniable benefits. For therapeutic applications, various types of liposomes have been synthesized. These different categories of liposomes vary in size, lipid content, number of lamellae, and surface changes.^23^ For biomedical applications of liposomes, various in vivo administration routes can be utilized including intravenous, oral, or topical.^24^ While showing considerable potential in clinical applications, liposomes are the earliest and most extensively studied nanocarriers for cancer drug delivery.

There are numerous ways to make liposomes, and each of them can be optimized to make them in the nanoscale range. Reverse-phase evaporation, injection techniques, electroformation microfluidic, thin-film hydration, detergent depletion, membrane extrusion, and heating are considered to be the conventional methods for liposome preparation.^25−30^ However, for uniform size formation of the liposome homogenization, sonication or ultrasonic irradiation techniques are employed.^30^ Recently, a more advanced “supercritical fluids” technique has also been developed for liposomal preparation.^30,31^ Regardless of the approach used, the lipid packing, fluidity, and phase transition temperature of phospholipid bilayers are all impacted by the size, type, and concentration of embedded nanoparticles (NPs) in the liposomes. The thin-film hydration approach is one of the most commonly used techniques for creating liposomes; it is a simple procedure that does not call for specialized tools, and it was the first technique to incorporate both hydrophilic and hydrophobic NPs in liposomes.^32,33^ Another alternate method to the thin-film hydration process used to quickly and easily make liposomes is the ethanol injection method. This technique, which is a member of the solvent injection family, involves injecting a water-miscible organic solvent that contains lipids into a sizable volume of aqueous buffer. Lipid nanoparticle have also been noncovalently modified with various open-chain and macrocyclic amphiphiles such as inorganic hybrids and amphiphilic *p*-sulfonatocalix[4]arenes.^34,35^

The primary drawbacks of liposomes are that they are rapidly eliminated from the blood (quick clearance) and these liposomes also cause the medications to be released prematurely before reaching the disease site.^36^ It is primarily caused by the blood’s adsorption of proteins, macrophage absorption, and the liposome’s instability of structure.^1^ Polyethylene glycol (PEG) is an excellent choice for making liposomes more stable and to make them circulate in the blood for prolonged periods.^37^ By having a connection to the outer structure, PEG can be exposed to the reticulon endothelial system (RES) less frequently and have a lower likelihood of being absorbed by the liver and spleen, and thus their rapid elimination from the body can be halted.^38^ Doxil was the first PEGylated liposomal formulation in 1995 which was approved in clinics for cancer treatment. Since then, various liposomal drugs including liposomal vaccines (Epaxal and Inflexal V, PEGylated liposomes (Lipodox), temperature-sensitive liposomes (ThermoDox), and cationic liposomes (EndoTAG-1) have been extensively studied for the delivery of therapeutics such as drugs and gene.^5^Table 1 details the list of liposomal-formulated drugs that have been studied for clinical trials.

**Table 1:** Liposome-Mediated Drug Delivery Studied under Clinical Trials

**Trade name**	**Therapeutic delivered**	**Disease**	**Route of administration**	**Nanoscale dimensions (nm)**	**Clinical trial Status**	**Ref**
DaunoXome	Daunorubicin citrate	Kaposi sarcoma	Intravenous	45	Approved	(39)
Doxil	Doxorubicin	Kaposi’s sarcoma	Intravenous	87	Approved	(40)
Evacet	Doxorubicin	Ovarian cancer	Intravenous	150	Approved	(41)
Lipo-Dox	Doxorubicin	Solid tumors	Intravenous	20	Approved	(42)
Nyotran	Nystatin	Solid tumors	Intravenous	110–135	Terminated	(43)
Alocrest	Vinorelbine	Solid tumors	Intravenous	100	Under study	(44)
Aroplatin	Cisplatin and its analog	Colorectal neoplasms	Intravenous/Intrapleural	-	Under study	(45)
ATI-1123	Docetaxel	Solid tumors	Intravenous	60–80	Under study	(42)
Atragen	Tretinoin	Solid tumors	Intravenous		Under study	(46)
Atu027	siRNA	Solid tumors	Intravenous	120	Under study	(47)
EndoTAG-1	Paclitaxel	Solid tumors	Intravenous	180–200	Under study	(48)
LEP-ETU	Paclitaxel	Solid tumors	Intravenous	150	Under study	(49)
LE-SN38	SN-38	Solid tumors	Intravenous	150–200	Under study	(50)
Lipotecan	Camptothecin	Solid tumors	Intravenous	180–200	Under study	(51)
MBP-426	Oxaliplatin	Solid tumors	Intravenous	180	Under study	(48)
MBP-Y005	Gemcitabine	Solid tumors	Intravenous	-	Under study	(52)
Myocet	Doxorubicin citrate	Breast cancer	Intravenous	190	Under study	(39)
NanoVNB	Vinorelbine	Colon cancer	Intravenous	95, 2	Under study	(53)

### 2.1 Application of Liposomes for Drug Delivery

**Figure 1:**
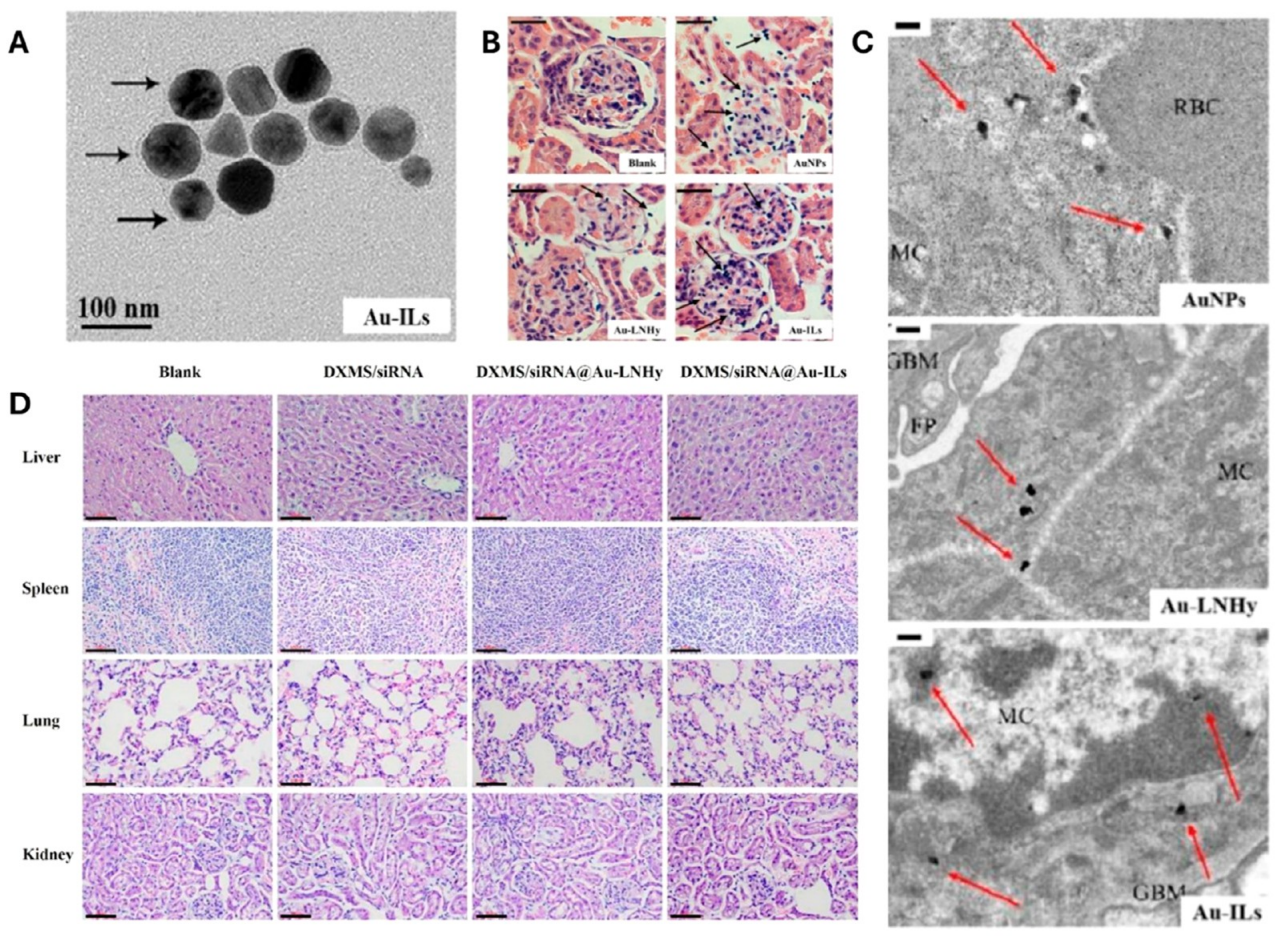
(A). Transmission electron micrographs of Au-ILs. (B) Au-IL nanoparticles had maximum distribution in the MC area compared to the other formulations. (C) Transmission electron micrographs of sections of kidneys treated with AuNPs, Au-LNHys, or Au-ILs. (D) HE staining assay of mouse heart, liver, spleen, lung, and kidney tissue sections after administration of DXMS/siRNA, DXMS/siRNA@Au-LNHy, or DXMS/siRNA@Au-ILs for analyzing *in vivo* toxicity. Adapted with permission from ref (63). Copyright 2021 American Chemical Society.

Numerous anticancer medications have an intermediate solubility, which allows them to easily segregate between the interior aqueous phase or the exterior of the liposome bilayer, leading to a fast release from the liposomes. The factors which leads to the fast release of drugs from liposomes are partitioning of drugs in the aqueous phase and the lipid bilayer, diffusion of drugs through the lipid bilayer due to its fluidic nature, outer part of the liposome bilayer presents a large surface area compared to the interior aqueous phase which provides more opportunities for the drug to be exposed to the surrounding environment, facilitating their faster release. Yet liposomes can effectively retain weak bases like daunorubicin by altering the inner pH of the liposomes or by forming complex molecular structures inside the liposomes.^54^ By loading pharmaceuticals to attain considerable intraliposomal concentrations of drugs beyond their solubility limitations, which enhances precipitation, or by packaging polyanions (for instance, dextran sulfate), it is possible to improve drug retention.^55^ Docetaxel is one example of a medication that may be transformed into a weak-base prodrug, enabling liposomal retention and encapsulation.^56^ Epirubicin, Dox, and daunorubicin are examples of antitumor anthracyclines that have extremely effective encapsulation. In contrast to free agents, whether solely or in pairing with other medications, liposomal anthracyclines have proven to be efficient and exhibit lower cardiotoxicity. The toxicological effects and efficacy of liposomal Dox vs traditional anthracyclines were examined in meta-analysis research.^57^ Both liposomal Dox and PEGylated liposomal Dox (PLD) demonstrated favorable toxicity profiles, with improved cardiovascular security as well as fewer alopecia, myelosuppression, vomiting, and nausea compared to conventional anthracyclines, thereby providing a better alternative for patients with risk factors for cardiovascular disease, and patients who have previously used anthracyclines.^57^ Recently choline phosphate (CP) lipids have been developed and found to have high efficacy for cancer therapy.^58^ Wang et al. demonstrated Dox encapsulated CP liposomes had higher uptake and accumulated in cells compared to phosphatidyl choline (PC) loaded Dox liposomes.^59^ This further showed significantly higher cytotoxicity in cancer cells and also inhibited growth of tumor. In a separate work CP liposomes were functionalized with PD-L1 antibody and encapsulated with Dox. This formulation showed enhanced melanoma cells penetrating ability with 100% tumor suppression rate.^60^ Li et al. further developed PD-L1 antibody conjugated CP-PC liposomes loaded with Dox to show enhanced antitumor ability in melanoma model with 94.4% tumor suppression rate in mice and 60% of the mice did not suffer from tumor recurrence.^61^ Prasad et al. have developed theranostic liposomes by encapsulating gold nanoparticles (AuNP) and graphene quantum dots (GQDs) for NIR active imaging and phototriggered chemotherapy. This liposomal formulation was functionalized with folic acid to target the breast cancer cells in vivo.^62^ Apart from chemotherapeutic applications, liposome have also been investigated for other diseases like Glomerulonephritis, which is a disease associated with kidney inflammation. Fang et al. have developed gold nanoparticle immunoliposomes (Au-ILs) and liposome-gold nanoparticle hybrids (Au-LNHy).^63^ These were encapsulated with Dexamethasone/TGFβ1-siRNA. In this study, Further to target the glomerular mesangial cell, α8 integrin antibodies were conjugated on the surface of the liposomes. Their observation showed effective targeting of the liposomes in the mesangial cells (MCs) within the glomerulus and further release of drug lead to suppressing local inflammation and fibrosis, resulting in improved therapeutic outcomes (Figure 1).^63^

### 2.2 Application of liposomes in gene delivery

Gene therapy has utilized viruses as an effective vector for gene transfection. Their high immunogenicity and difficult preparation procedure, however, significantly restrict their potential use. At the same time, DNA transfection has restricted use due to the poor transgenic expression capacity and immunogenicity of plasmid DNA.^64^ Thus, the current approaches involve encapsulating mRNA in liposomes for delivering systemic tumors and using mRNA rather than DNA for transfection.

Drug resistance in cells can be decreased by carrying genes and medications as a combination.^65^ For the transport of siRNA and anticancer medications, Shim et al. developed a liposome-based on trilysinoyl oleamide.^66^ Following being modified with PEG, it was discovered that intravenous injection of this liposomal formulation drastically decreased the expression of the human Mcl1 protein in KB-xenografted tumor tissue. DOX-encapsulated liposomes boost the anticancer action at the same time, and following intravenous administration, they saw a considerable decrease in tumor size.^67^

Since liposomes that are cationic are biodegradable and contain a strong positive charge, gene delivery techniques frequently employ them. Though cationic liposomes may efficiently encapsulate RNA and improve load efficiency, their positive charge will cause toxicity in living cells. Due to the electrostatic contact among liposomes and plasma proteins, this results in liposomes being mostly taken up by the liver and kidneys, which ultimately restricts their use in the human system.^68^

**Figure 2:**
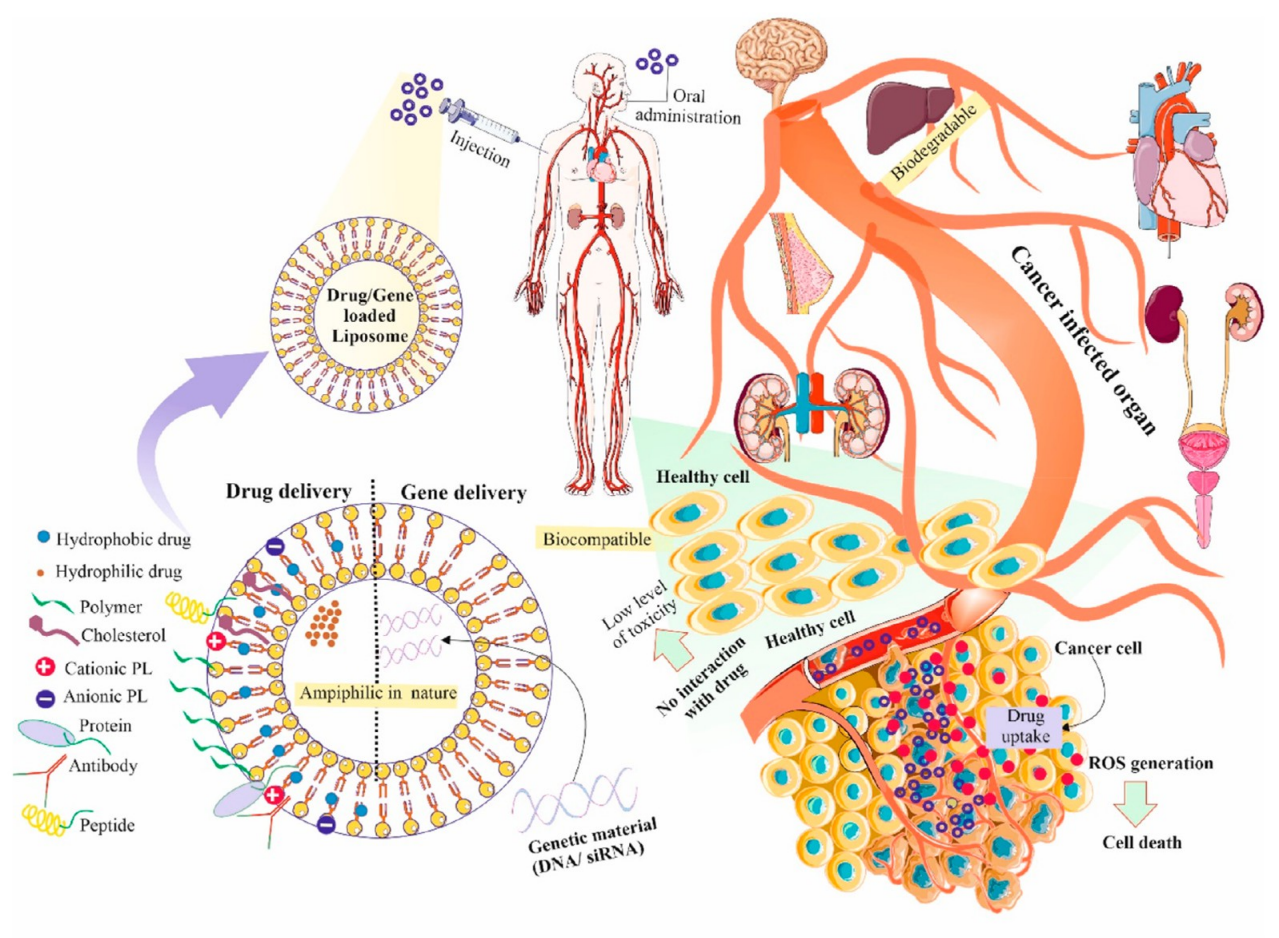
Representation of liposomes used for several types of drug delivery as well as gene delivery due to their unique properties. A wide variety of hydrophilic and hydrophobic diagnostics or therapeutic agents are easily encapsulated with a sustained release capability. Parts of the figure were drawn using pictures from Servier Medical Art. Servier Medical Art by Servier is licensed under a Creative Commons Attribution 3.0 Unported License.

Hence, neutral or PEGylated liposomes are employed for the transport of genes. Trang et al. created a neutral liposome emulsion NLE to transport Let-7 and MiR-34a.^69^ The lung showed the greatest concentration of the antisense oligonucleotides (ASOs) and DOX that were codelivered via PEG-modified cationic liposome.^70^ This liposome demonstrated a noticeably strong suppression of tumor regression. However, due to the accumulation of this liposome in the lungs, the application is limited for treating other malignancies. Although this unquestionably offers a fresh perspective and renewed hope for liposome-based gene delivery. Liu et al. demonstrated malate dehydrogenase, DSPE-PEG 2000, and cholesterol-based hypoxia-responsive ionizable liposome for the delivery of polo-like kinase 1 siRNA into glioma cells.^71^ Using their approach, the development of glioma cells was found to be significantly inhibited by this liposome. Thus, liposome demonstrates the potential for both drug and siRNA delivery in cells from various tissue origin (Figure 2).

## 3 Solid Lipid Nanoparticle

Second-generation lipid nanocarriers, also known as SLNs, are spherical colloidal nanoparticles stabilized by surfactants and have a solid lipid core made of waxes, triglycerides, and fatty acids. Some of the commonly used lipids used for synthesis of SLNs are stearic acid, palmitic acid, and glyceryl monostearate. They are primarily recognized for their biocompatibility, increased sensitivity to lymphatic absorption, and sustained drug release. These SLNs typically range in size between 50 and 100 nm. First discovered in 1991, these SLN quickly attracted the interest of scientists due to their promising application as drug delivery systems for compounds with poor solubility and limited bioavailability. SLNs are a colloidal dispersion of nonpolar lipids, such as triglycerides and fatty acids, which are solid at physiological temperatures as well as room temperatures.^72,73^ There are several preparation methods for producing the SLNs, such as high-pressure homogenization, high-speed stirring-ultrasonication, microemulsion, solvent emulsification-diffusion, solvent emulsification-evaporation, double emulsion, phase inversion temperature, membrane contactor, supercritical fluid-based, coacervation, and solvent injection.^74−80^ So far SLNs have been widely used for the delivery of chemotherapeutic drugs into tumors.^3,81^ Apart from standard chemotherapeutic delivery, SLN has also been utilized for magnetic resonance imaging using superparamagnetic iron oxide encapsulated SLN,^6^ positron emission tomography using technetium-99 (99 mTc) or 64Cu encapsulated SLN,^82^ and quantum dots encapsulated SLN for near-infrared imaging of cancer cells.^9,10^

The primary advantage of employing SLNs in therapeutic applications is that they are composed of safe and FDA-approved ingredients, which makes the carriers nontoxic. The major technique for synthesizing SLNs involves creating a pre-emulsion between the solid lipid and the surfactant, also known as an emulsion, and then reducing the size of the mixture using techniques like homogenization and ultrasonication. The common surfactants used for SLN synthesis are polysorbate 80 (Tween 80), poloxamer 188, polysorbate 20 (Tween 20), and phosphatidylcholine. The delivery of biomolecules by SLN has shown encouraging results in a variety of industries, including the pharmaceutical, cosmetic, and biological research domains.^83^ Solid lipids (at room and physiological temperatures) stabilized with surfactants and cosurfactants that may ensure particular qualities are used to create SLN formulations. One of the promising nanocarriers to overcome the limitations of poorly absorbed medications and increase their bioavailability is SLNs, which are absorbed and transported via transcellular and paracellular pathways. As highlighted earlier, it is possible to encapsulate bioactive lipophilic compounds into the solid lipid matrix and release them in a controlled way.^8,84^ This drug entrapment process in the core matrix is influenced by several factors, including the types of solid lipids used, the solubility of the drug in the chosen lipids, manufacturing processes, and polymorphism criteria in the lipid matrix.^85^

Although solid lipid constitutes the majority of SLNs, degradation, and instability may become an issue. The minimal drug loading potential, the kinetics of the delivery process, the coexistence of various lipid modifications and colloidal species, and high pressure-induced drug degradation are some of the factors that need to be taken into account. Due to their high brittleness, large molecular weight substances like DNA, albumin, and dextrose must be integrated into SLNs using a different strategy. Because dynamic processes are essential for drug stabilization and release, the mere existence of diverse heterogeneous entities is insufficient to characterize the structure of colloidal lipid phase separation. The kinetics of distribution mechanisms must therefore be taken into consideration. Since they are composed of solid lipids, SLNs are excellent carriers for lipophilic medications, but creating one that can also transport water-soluble compounds is still a long way off.^2^ Due to their lack of affinity for the lipid matrix, water-soluble molecules have a strong propensity to partition into the outer aqueous phase throughout the production process.^86^

Hu et al. assessed the stability of SLNs in the simulated gastric media.^87^ In contrast to SLNs lacking poloxamer 188, which exhibited considerable and immediate aggregation following incubation in the gastric medium, SLNs containing poloxamer 188 demonstrated a protective coating effect, and no aggregation was observed.^87^ Poloxamer-coated SLNs did not alter particle size, and there was very little lipid breakdown in the stomach media. Exciting findings by Hu et al. also showed that using SLNs dramatically increased the absorption of the payload, i.e., all-trans retinoic acid along with performance enhancement of poorly soluble drugs by reduction of particle size. Additionally, drug surface area and saturation solubility are improved by SLN encapsulation.^87^

### 3.1 Biomedical Application of Solid Lipid Nanoparticles

**Figure 3:**
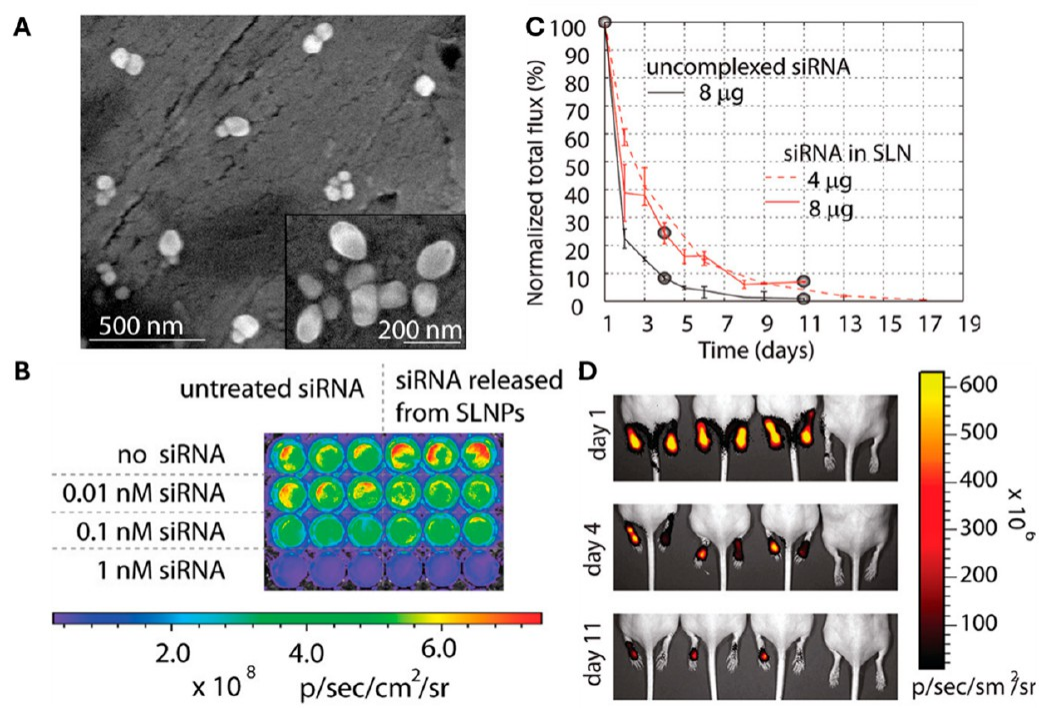
(A) Scanning electron microscope images of SLNPs. (B) In vitro siRNA activity as compared between untreated (free siRNA) and SLNs encapsulated siRNA activity on Human 293FT cells that were cotransfected with pTD138 (a plasmid that expresses click beetle luciferase CBL). (C) Normalized total flux versus time shows *in vivo* release of unencapsulated siGLO Red (black solid line) and siGLO Red encapsulated in SLNPs (red lines); (D) *In vivo* activity of siRNA released from SLNs as from red fluorescence of siGLO Red in mice paws after day 1, day 4, and day 11 of administration. Adopted with the permission from ref (91). Copyright 2011 American Chemical Society.

The pattern of biodistribution of anticancer medications in the body may be changed by SLNs. Biodistribution research predicts potential drug adverse effects on other sections of the body and demonstrates how pharmaceuticals affect tumors and organs. According to Liu et al., when compared to quercetin suspension, quercetin-loaded SLNs may considerably accumulate in various organs following oral treatment.^88^ In comparison to the control, the impact of RGD-SLNs on MDA-MB-231 cell invasion through Matrigel was assessed. Each nanoparticle formulation was present when cells were allowed to invade toward an FBS gradient through a Matrigel-coated trans well filter. All four RGD-decorated nanoparticle formulations significantly decreased invasion (*p* < 0.05), and the inhibitory effect was stronger as RGD concentration increased.^89^ Apart from therapeutic activity SLNs have been utilized for in vivo imaging applications. Mannucci et al. developed a SLN coloaded with cardiogreen (CG) which can be detected using an optical imager and rhodamine (RH) for detection using fluorescence microscopy. The in vivo administration of the formulation showed the SLNs accumulation in hepatocytes without any toxicity as observed from tissue sections.^90^ SLNs have also been utilized for nucleic acid delivery *in vivo*. Lobovkina et al. developed siRNA loaded Tristearin SLNs.^91^ It was observed that the siRNA has a more stable and sustained release compared to free siRNA. Further its gene silencing efficacy upon release from SLNs was significantly high in both the cell line as well as *in vivo* (Figure 3).^91^

## 4 Liquid Lipid Nanoparticles

Lipid nanomaterials with a nonlamellar lyotropic liquid crystal (LLC), primarily made of lipids with amphiphilic properties have shown great promise as the next generation of nanomedicines. They resemble liposomes but contain intricate, nonlamellar nanostructures in two and three dimensions, such as inverse hexagonal, cubic mesophases, and the short-range structure of interconnected water channels. These structures are frequently referred to as “hexosome”, “cubosome”, and “spongosomes” to indicate the interior hexagonal mesophases, inverse cubic, and intermediate mesophase between the lamellar and cubic phase, respectively.^92^ Cubosomes and hexosomes have been demonstrated to be successfully conjugated with antibodies, making them more desirable for the targeted administration of drugs.^93^ Additionally, due to LLC versatility, hydrophilic and hydrophobic small molecule medicine,^93,94^ nucleic acids,^18,95^ peptides, proteins,^96,97^ and imaging agents^98,99^ can be transported using them as nanocarriers.

Phytantriol (PT), Monoolein, sometimes referred to as glycerol monooleate (GMO), Poloxamer 80, and Pluronic F127 (also known as Poloxamer 407) are the lipid polymers used to prepare LLC NPs. 70% of the in vivo studies that used GMO serving as an anchor matrix for LLC NPs for the delivery of drugs favored it. This is due to the biocompatibility and nontoxic property of GMOs, which favors their use as a food enhancer.^100,101^ PT is resistant to hydrolysis and enzymatic breakdown because it does not include any ester or unsaturated linkages. PT is a frequent element in personal care and beauty products, making it economically available and cost-effective.^102,103^ For the stabilization of hexosomes and cubosomes, additional stabilizing agents, such as citron, various other Pluronics (such as F128), PEGylated lipids, and β-casein, have been used in combination with Pluronic F127.^104−106^

It was discovered that mPEG-lipid conjugates might exhibit modulatory impacts on nanoparticle-mediated stimulation of complement when used as stabilizing agents. It has been noted that TPGS-mPEG2000 is an especially desirable lipopolymer for successfully preventing the activation of complement among the various mPEG-lipid conjugates.^107^

### 4.1 Cubosomes

There are several types of cubosomes such as bicontinuous, imaged, hexosomes, Janus, and hybrid cubosomes, among which the widely recognized and extensively researched variety of cubosomes are bicontinuous ones. These are made up of two lipid bilayers that interpenetrate and organize themselves in a periodical cubic matrix. The interior structure of the cubosomes is bicontinuous because the lipid bilayers provide an ongoing system of water channels. Bicontinuous cubosomes and imaged cubosomes are similar; however, imaged cubosomes have a more complicated internal structure. Imaged cubosomes have more internal compartmentalization because several linked lipid bilayers are arrayed in a recurring cubic lattice. In order to improve their durability, loading capability, or specialized capabilities, hybrid cubosomes mix in additional elements like polymers or nanoparticles to the lipid matrix. By combining the benefits of several materials, these hybrid constructions can be advantageous.

The most common surfactant used in cubosome preparation is Poloxamer 407 (P407), a poly(ethylene oxide)-99, poly(propylene oxide)-67, and [PEO99-PPO67-PEO99] triblock copolymer.^7^ Its PPO portions are found either directly on the exterior of the cubosomes or inside the bilayer structure, while the PEO chains are made accessible to the water that surrounds them. Smaller particles were more successfully produced at the higher P407 concentrations, although this circumstance also favors vesicular particle creation as opposed to the desired nanostructured substances with the cubic matrix. When it comes to cubosomes, the stabilizing process of P407 appears to be distinct from that of straightforward dispersions like emulsions. The stabilizer affects the arrangement of the scattered particles and controls their phase behavior in cubosomes. Particularly, a sufficient concentration of P407 ensures the existence of the P-type cubic phase, which is in charge of forming a stable colloidal dispersion.

#### 4.1.2 Methods for Preparation of Cubosomes

**Figure 4:**
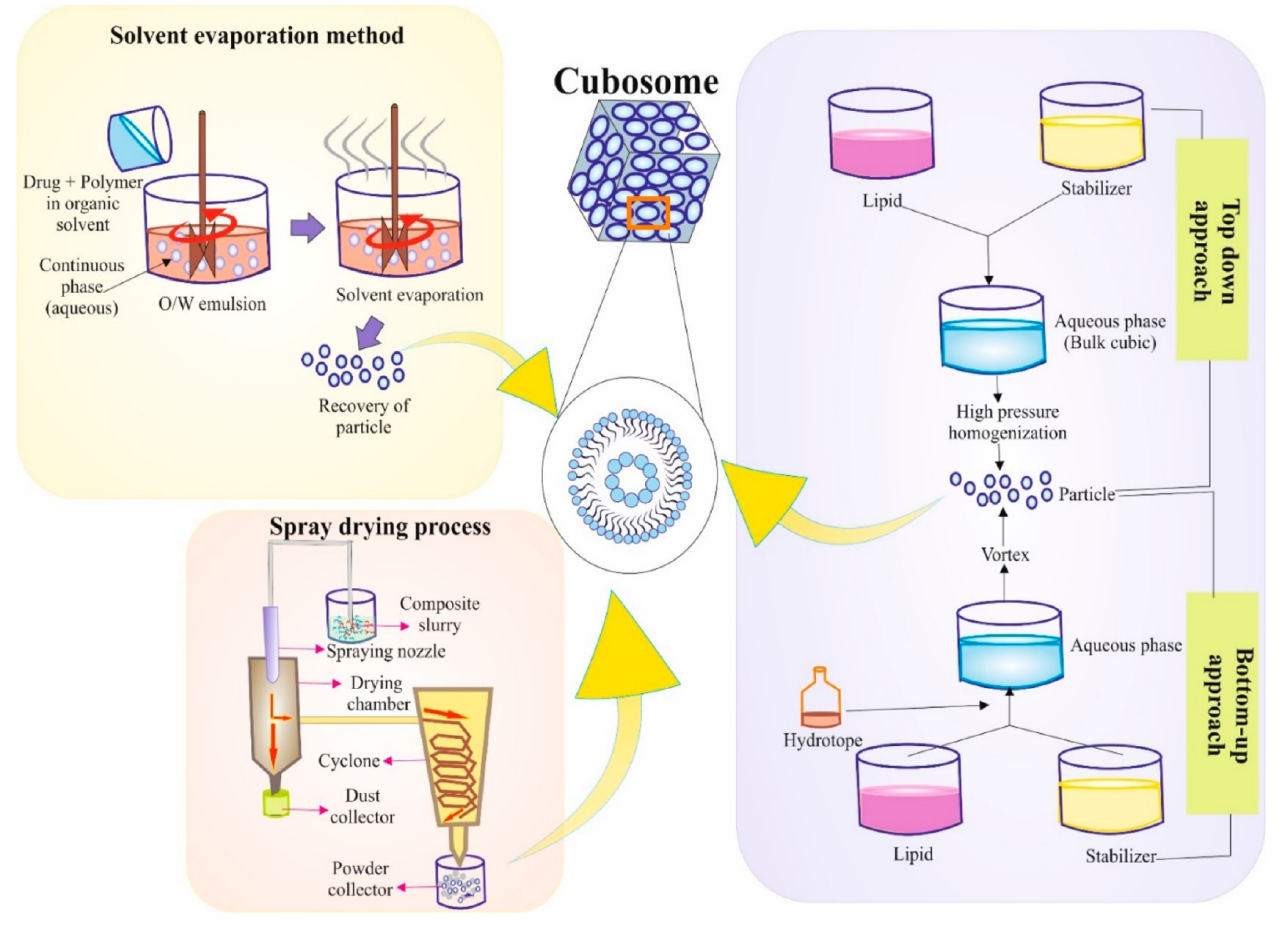
Schematic representation of cubosome preparation by different methods. Here the solvent evaporation method, spray drying process, and top-down and bottom-up mechanisms are elaborated. Parts of the figure were drawn using pictures from Servier Medical Art. Servier Medical Art by Servier is licensed under a Creative Commons Attribution 3.0 Unported License.

Method for the preparation of cubosomes include the bottom-up method, top-down method, spray-drying method, and solvent evaporation. Lipid molecules self-assemble into cubosomes via the bottom-up approach (Figure 4). It depends on the lipid’s capacity to spontaneously coalesce into structured arrangements in an aqueous environment. Typically, an organic solvent is used to dissolve a lipid combination at the beginning of the procedure. The organic solvent is subsequently eliminated by evaporation or by another similar process, leaving a lipid coating in its place. An aqueous solution is used to hydrate the film, and then mechanical agitation—such as vortexing or sonication—is used to encourage the formation of cubosomes from lipids. The top-down approach entails mechanically disrupting a bulk cubic phase to produce cubosomes. This technique involves applying mechanical forces, including high-pressure homogenization or sonication, to a cubic phase made of a lipid mixture. Cubosomes are created when these pressures divide the main cubic phase into smaller pieces. When beginning with a ready-made bulk cubic phase, such as a liquid crystalline gel, the top-down approach is frequently utilized. Dry cubosome powders are made using the spray-drying procedure. This technique involves employing a spray nozzle to atomize cubosome-containing lipid dispersion into tiny droplets. The droplets are then exposed to a stream of hot air, which causes the solvent to quickly evaporate and create dry cubosome particles. Cubosomes may be handled easily and stored for a long time using the spray-drying technique. Cubosomes are frequently produced via the solvent evaporation process, sometimes referred to as the solvent diffusion method or solvent removal method. This approach involves gradually adding an organic solvent-dissolved lipid combination to an aqueous solution while stirring continuously. Lipids self-assemble into cubosomes as a result of the organic solvent diffusing into the aqueous phase. To fine-tune the cubosome structure, the solvent evaporation approach is frequently supplemented with other processing stages like sonication or filtering (Figure 4).

### 4.1.3 Drug Loading Techniques in Cubosomes

Cubosomes could be loaded with small peptides, molecule drugs, bioactives, or biologics to function as a possible drug delivery system. The three primary methods of encapsulating the payload include localizing the medicine inside the water channel in the cubic phase, attaching it to the lipid membrane, and loading it between the lipid bilayer.^108^ The therapeutic agent might be added to the molten lipid or lyophilized with the film of lipids before dispersion.^4,98,109^ As an alternative, the incubation process may also be used to load drug moieties into cubosomes that had already formed following dispersion.^110,111^ Furthermore, these cubosomes are created using mono or dual lipid compositions, primarily monoolein, and phytantriol.^111^ Cubosomes have been used in several experiments to administer drugs, with encapsulating effectiveness varying from 71 to 103%.^4,99,112^

#### 4.1.4 Routes for Cubosome Administration In Vivo

Cubosomes have been administered using oral as well as intravenous routes.^104,113,114^ The improved bioavailability and sustained release of several drugs, involving doxorubicin, cinnarizine, and 20(S)-protvopanaxadiol, loaded cubosomes, in the production of nanoparticles for use in oral drug delivery.^115−117^ The examined nanoparticles were stabilized with Pluronic F127 and were designed around either monoolein (MO) or phytantriol (PHYT). For instance, Swarnakar et al. when gave doxorubicin-loaded PHYT cubosomes orally to rats, the FDA-approved formulation adriamycin, which was given intravenously, showed higher bioavailability, a lower degree of cardiotoxicity, and improved antitumor effectiveness.^118^ A prolonged circulatory half-life and a better tumor buildup of nanoparticles via a stronger penetration and retention (EPR) effect were credited for this improved oral doxorubicin administration.^118^

Following intravenous treatment of mice, NIRF imaging was used to examine the real-time distribution of the cubosomes. It was discovered that the mice’s liver and spleen had accumulated levels of the supplied nanoparticles for up to 20 h after delivery.^119^ Compared to equivalent non-PEGylated cubosomes and plain paclitaxel, radioactive labeling (99 mTc-Technetium radionuclide) of PEGylated cubosomes that are loaded using paclitaxel is not solely correlated with an increased level of safety but also accounts for an enhanced tumor accumulation and improved circulation time by EPR.^120^ The internalization of the non-PEGylated nanoparticles inside the tumors by other nonspecific effects than EPR was responsible for the reported tumor growth inhibition.

## 5 Advantages of Cubosomes over Other Nanocarriers

The most important advantages of cubosomes are their biocompatibility, capacity to be loaded with a variety of drugs, and ease of use. These cubosomes are considered to be more stable than liposomes for having liquid crystalline membrane design along with a stronger potential to surround and encapsulate hydrophobic chemotherapeutic drugs which may provide continuous release of drugs over long periods.^121^ The major benefit of this cubosome over any other nanoparticle including liposome that it may allow more hydrophobic drugs with a larger hydrophobic area still allowing for hydrophilic drug loading.^122^ Cubosomes are regarded as potential carriers for various routes of administration because of unique properties such as thermostability, adhesion, the ability to encapsulate drugs, and the capacity to sustain release.^123^ According to a report of a prior study that compared cubosome and liposome entrapment efficacy, cubosomes which was prepared by using Phytantrio showed a greater result, owing to deeper penetration of the curcumin molecule into the hydrophobic area.^124^ It is vital to note that stabilizers may interact with particle interior structures. For example, the generated structures in monoolein cubosomes assembled with a low concentration of F127 exhibit a tetrahedral arrangement of short rods of the minority component (Pn3m shape).^125^ Another major consideration in the development of nanoparticles is toxicity regulation. Cubosome cytotoxicity is affected by a variety of parameters, including internal nanostructures, lipid chemistry, and the type of stabilizers. According to a study by Fornasier et al., polyphosphoester (PPE), a structural equivalent of classical F127, was used to create cubosomes. The formulated cubosomes were found to be much less harmful than carriers made with F127 evaluated against HEK-293 and HUVEC. The poly(phosphoester)-based formulation was also shown to have a high hemocompatibility in contrast to cubosomes made using F127, which exhibit mild cytotoxicity toward erythrocytes.^126^ Cubosomes loaded with multidrugs that target endoplasmic reticulum stress as a potential new therapeutic approach for treating neuronal degeneration.^127^ The benefits of cubosomes include the solubilization of lipophilic, hydrophilic, or amphiphilic pharmaceuticals, the prolonged release of integrated medications, adhesion, drug protection from degradation, and the nontoxic nature of the cubosomes.

## 6 Applications of Cubosomes in Various Biomedical Fields

### 6.1 Antifungal Application

**Figure 5:**
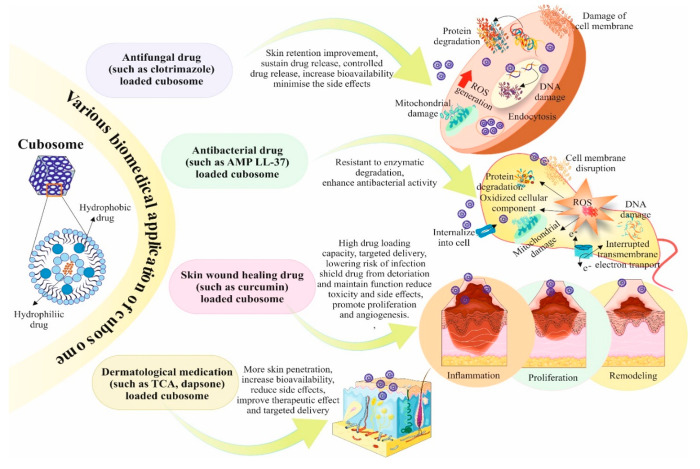
Various biomedical applications of cubosomes are described. Encapsulating drugs in cubosomes enhances antifungal, antibacterial, wound healing, and dermatological medication by improving skin retention, sustaining drug release, resisting enzymatic degradation, increasing drug loading capacity, targeting drug delivery, increasing bioavailability, and many more. Parts of the figure were drawn using pictures from Servier Medical Art. Servier Medical Art by Servier is licensed under a Creative Commons Attribution 3.0 Unported License.

For the topical treatment of fungal infections, clotrimazole is the commonly prescribed medicine. The ineffectiveness and restricted local availability of clotrimazole are caused by its poor skin retention and limited water solubility. Clotrimazole’s cubosomal formulation demonstrated improved skin retention. The ability of cubosomes to pass through the skin corneocytes via the paracellular pathway causes the first phase to be characterized by rapid drug release, and the second phase is characterized by sustained drug release because cubosomal nanoparticles can create a depot in the lipid layer of the stratum corneum^128^ (Figure 5).

Due to the high drug-loading capacity of cubosomes, antifungal medications can be effectively encapsulated inside their lipid bilayers. As a result, drug concentrations are elevated at the infection site and drug delivery is improved. Cubosomes can also be developed to have controlled drug release characteristics, allowing for the continuous and protracted release of antifungal medicines. By lowering the frequency of dose and perhaps enhancing patient compliance, this controlled release contributes to the maintenance of effective medication concentrations over a prolonged period. Additionally, cubosomes can be altered with targeting ligands to enable tailored distribution to the fungus infection site. With little adsorption in healthy tissues, this targeted strategy increases the bioavailability of the drug at the infection site, minimizing adverse effects and increasing treatment efficacy (Figure 5).

### 6.2 Antibacterial Application

In order to protect against microbial infection, the skin produces fatty acids and sebaceous secretions, acting as a barrier. Bacteria can enter tissues through skin outbreaks caused by cuts, surgeries, needle injections, burns, and scrapes.^129^ As a therapeutic approach against bacterial infections, cubosomes have been employed for the delivery of antimicrobial peptide (AMP) LL-37. In comparison with unloaded LL-37 that was not enclosed in the cubosome, LL-37 encapsulated in the cubosome was resistant to enzymatic degradation, and the bactericidal effect was maintained regardless of enzymatic exposure. LL-37 encapsulated cubosomes were shown to be the most efficient in inhibiting bacterial infection having no possibility for skin irritation in the acute wound infection scenario.^130^ In another study done by Meikle et al., Gram-negative bacteria and Gram-positive were used as test subjects for the activity of silver nanocrystals encapsulated into cubosomes.^131^ When contained inside the cubosomal framework, the silver nanocrystals’ antibacterial activity had a much-enhanced effect on the bacterial cells which serves as an exploratory platform to demonstrate the promising therapeutic ability of cubosomes^131^ (Figure 5).

### 6.3 Application in Wound Healing

Skin wound healing is a cellular repair procedure that involves growth factors, cytokines, and cell-to-cell contact that encourage lesion closure. Cubosomes can efficiently encapsulate therapeutic substances like growth factors or antimicrobials within their lipid bilayers, with high drug loading capacity. This makes it easier to distribute these medications to the wound site in a targeted and regulated manner, accelerating the healing process and lowering the risk of infection. The medicinal chemicals that are contained in cubosomes are stabilized by the lipid bilayers, which also shield them from deterioration and maintain their function. The long-term effectiveness of the administered medicines throughout the wound healing process depends on this stability. When administered to wounds, cubosome’s biocompatibility reduces the possibility of toxicity or side effects. Cubosomes can promote cell proliferation, angiogenesis (the development of new blood vessels), and extracellular matrix production, all of which are crucial for wound healing, by delivering these elements right to the wound site. As studied by Shetty et al., curcumin (CUR) boosts collagen production and lowers keratinocyte apoptosis, which promotes wound healing and promotes fibroblast proliferation.^132^ In comparison with unloaded curcumin, cubosome hydrogel that was loaded with CUR demonstrated improved permeability and a 3.8-fold higher retention. Additionally, a larger zone of inhibition against bacterial cells was seen in the cubosome formulation which demonstrates the potential of cubosome formulations for drug delivery^133^ (Figure 5).

### 6.4 Dermatological Application

To treat bacterially induced skin infections, triclosan (TCA) is utilized. The ability of TCA-loaded cubosomes to penetrate skin was tested by Kwon et al.^134^ When transported via the epidermis into the aqueous receptor solution, the cubosomal suspension showed more skin penetration than the unloaded TCA. As a result, TCA-containing cubosomes are successfully employed in the creation of antiacne cosmetics.^134^ Acne is treated with the anti-inflammatory medication “dapsone”. Enzymes like hydroxylation and acetylation transform dapsone into dapsone hydroxylamine, which has a low bioavailability and side effects. In a study by Nithya et al., dapsone was encapsulated within cubosomes made of GMO and P407. The transdermal flow value of dapsone contained in cubic lipid particles was higher and showed that larger concentrations would improve the therapeutic effect at the targeted site^135^ (Figure 5).

### 6.5 Application of Cubosome in Cancer Therapy

Cubosomes have shown promise as vehicles for the delivery of drugs that target tumors. Utilizing cubosomes for tumor-targeted delivery entails making use of these nanoscale structure’s special traits to transport therapeutic drugs directly to tumor cells while limiting their influence on healthy tissues. The fundamental benefit of employing cubosomes for this objective is their capacity to transport a variety of therapeutic substances, including imaging agents, genes, and anticancer medications. Blood vessels in tumors are frequently aberrant and unstable. Cubosomes may utilize the leverage of this EPR phenomenon by selectively clustering in tumor tissues because of their nanoscale diameter. The Enhanced Permeability and Retention (EPR) effect is used by cubosomes as tumor-targeted drug delivery mechanisms to improve the buildup of therapeutic drugs selectively at the tumor site. Solid tumor blood vessels tend to be leakier and more permeable compared to healthy tissue. This aberrant tumor vasculature causes cubosomes and other nanoscale particles to build up inside the tumor tissue via the EFR effect.

**Figure 6:**
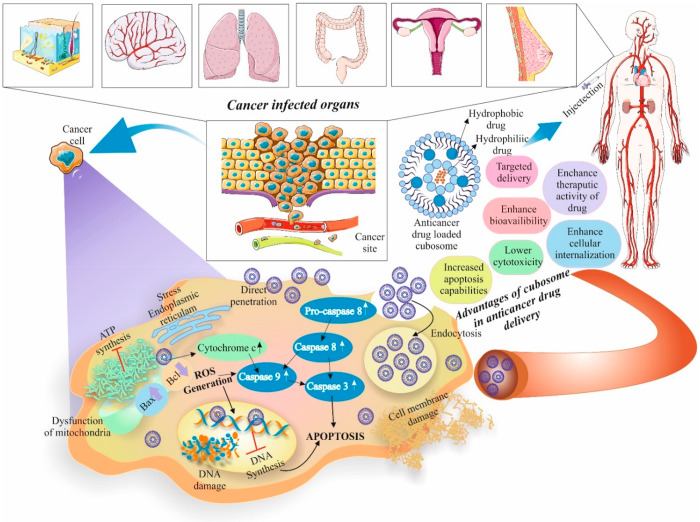
Detailed mechanism of therapeutic loaded cubosomes for various types of cancer treatment including brain, skin, lungs, colon, and breast cancer. Therapeutic activity was enhanced for drugs loaded on cubosomes, with targeted drug delivery and lower toxicities detected as well as increased apoptosis in cancer cells. Parts of the figure were drawn using pictures from Servier Medical Art. Servier Medical Art by Servier is licensed under a Creative Commons Attribution 3.0 Unported License.

Cubosomes are an ideal candidate for tumor-targeted drug delivery since using a combination of both active and passive targeting techniques can increase their total tumor-targeting accuracy. Since they have their lipid makeup, cubosomes may contact and even fuse with the exterior of the cell, allowing the material inside to be transferred directly into the cytoplasm of the cell. Drug efflux pumps are located on the cell membrane, that are responsible for ejecting out drugs from cytoplasm and thus help gaining drug resistance by various cancer cells. The ability of cubosomes to bypass the efflux pumps located on the cell membrane lowers the likelihood of drug ejection. Cubosomes have been explored as efficient therapeutic delivery agents in several cancers as detailed in the next section (Figure 6). Table 2 details the therapeutic application of cubosomes in various cancer models both *in vitro* and *in vivo*.

**Table 2:** Drugs and Biomolecules Used for Cancer Therapeutics and Delivered via Cubosome Nanocarriers

**Drug used**	**Cell line/cancer model**	**Dimension of cubosome (nm)**	**Targeting**	**Ref**
Gambogenic acid	SMMC-7721 (Hepatocellular carcinoma)	148	No	(136)
5-Fluorouracil	MDA-MB-231 (Breast cancer)	187.2	No	(137)
Albendazole	HepG2 (Hepatocellular carcinoma)	48.17 ± 0.65	No	(138)
Imatinib mesylate	Hep G2 (Liver cancer)	130.7 ± 2.92	CD44 via hyaluronic acid	(139)
Bedaquiline	A549 (Lung cancer cells)	150.2 ± 5.1	No	(140)
Paclitaxel	Hela (Cervical cancer)	138.7 ± 6	Biotinylated cubosomes	(98)
Metformin	Hct-116 and Caco-2 (Colorectal cancer)	110–160	No	(141)
siRNA	CHO (Chinese hamster ovary)	332	No	(142)
Doxorubicin, 213 Bi	HeLa (Cervical cancer)	160 ± 10	No	(143)
Curcumin and fish oil antioxidant	SH-SY5Y (Neuroblastoma)	100 nm and 400	No	(144)
Docetaxel	Hela (Cervical cancer)	174	Folate via folic acid	(112)
Naproxen Na	Hela (Cervical cancer)	200 ± 39	No	(145)
NaYF4:Er3+,Yb3+ UCNPs	SKOV-3 (Ovarian cancer), MeWo (Melanoma granular fibroblasts)	163 ± 7	Folate via folic acid	(146)
5-fluorouracil	HepG2 (Hepatocellular carcinoma)	105.7075.47	No	(147)
Bambusae Caulis	Raw 264.7 (Mouse Leukaemia)	166–179	No	(148)
5-FCPhy	MCF7 (Breast cancer), PC3 (Prostate cancer)	164	No	(149)
Lumefantrine	A549 (Lung cancer)	259.4 ± 19	No	(150)
Brucea javanica Oil and Doxorubicin	MCF-7 (Breast cancer)	<200	No	(151)
Ce6 or TPP-Mn	Me45 (Skin melanoma), MeWo (Melanoma fibroblasts)	between 130 ± 1 and 162 ± 4	No	(16)
Curcumin	Hela (Cervical cancer)	100–300	No	(152)
Rapamycin	NK-92 (Natural killer cell)		No	(153)
Lipopeptide	MCF-7 (Breast cancer), 161Br (Skin fibroblasts)		No	(154)
dsDNA	CHO (Chinese hamster ovary)	∼250–420	No	(155)
Capecitabine, 5-FCOle	MDA-MB-231, 4T1 (Breast cancer)	255	No	(156)
Copper-organo complex	LS174T (Colorectal cancer)	141	Carcinoembryonic antigen via Affimer protein	(13)
Copper-organo complex	MDA-MB-231 (Breast cancer), HT29 (Colorectal cancer)	152	CD44 via hyaluronic acid	(12)

#### 6.5.1 Skin Cancer Therapy

Skin cancer is associated with several types of carcinomas, such as basal cell carcinoma, cutaneous squamous cell carcinoma, and melanoma. Drug resistance arises in skin cancer as a result of either acquired resistance during cytostatic therapy or innate resistance.^157^ Thus, to overcome the challenges in treatment, cubosomes have been employed to overcome resistance and boost the quantity of medications reaching tumor locations.^16^

A commonly used chemotherapy drug for skin cancer is paclitaxel (PTX). It belongs to the taxane class of medications and has proven to be highly effective in treating a variety of skin cancer situations, including nonmelanoma and melanoma skin cancers (basal and squamous cell carcinoma). Cubosomes serve as a potential carrier for the delivery of paclitaxel (PTX) in the treatment of skin cancer. Zhai et al. performed a study to confirm if paclitaxel (PTX)-loaded cubosomes could prevent the proliferation of skin cancer in vivo.^15^ Following a two-week course of therapy, the research team found that PTX reduced the proliferation of the A431 tumor by reducing the tumor volume from 360 mm^3^ to 250 mm^3^ but only free-PTX treated group when compared to those mice injected with PTX-loaded cubosomes had a final tumor of 160 mm^3^ that was reduced by 0.7 times. This observation was justified based on whole-body biological distribution, which concluded that PTX-cubosome concentrates significantly in tumor sites when compared with PTX-free.^15^ As per the report of a prior study, the antimelanoma drug resveratrol, with low bioavailability and therapeutic activity, was loaded into the cubosome to increase its activity.^158^

#### 6.5.2 Glioblastoma Multiforme Therapy

The therapy of glioblastoma multiforme (GBM) is challenging due to the blood-brain barrier which prohibits drugs from reaching the tumor site. Flak et al. used cubosomes as drug delivery vehicles to efficiently transport therapeutic medicines to the location of the brain tumor.^159^ In their work, hydrophobic molecules, like AT101 are used as the therapeutic molecule for improving its bioavailability by encapsulating in the lipid membrane of cubosome. Unconjugated AT101 results in binding affinity to proteins, which is associated with reduced efficacy of AT101, whereas cubosome conjugated AT101 showed minimal protein binding and, as a result, had a higher therapeutic effect. As observed in the study, the enhanced cytotoxicity response to GMO-AT101 cubosomes may perhaps be a result of the strong internalization connected to endocytic channels.^159^

#### 6.5.3 Lung Cancer Treatment

Bedaquiline (BQ) is a member of the diarylquinoline class of drugs with anticancer characteristics that is designed for the treatment of lung cancer cells. The lipid bilayers of cubosomes enclose BQ, creating BQ-loaded cubosome (BQLC).^140^ Cubosomes’ tiny size enables them to preferentially aggregate after systemic delivery in the lung tumor tissues. In addition, surface alterations of cubosomes with ligands or antibodies particular to the receptors on lung cancer cell surfaces can improve active targeting. By enabling selective absorption of BQLC by cancer cells, this alteration improves drug delivery to the tumor location while lowering the exposure of healthy lung tissues. As studied by Patil et al, in nonsmall cell lung cancer (A549) cells, the BQLC demonstrated better cellular internalization and cytotoxicity with a 3-fold lower IC50 compared to free BQ after 48 h of treatment.^140^

#### 6.5.4 Colorectal Cancer Therapy

**Figure 7:**
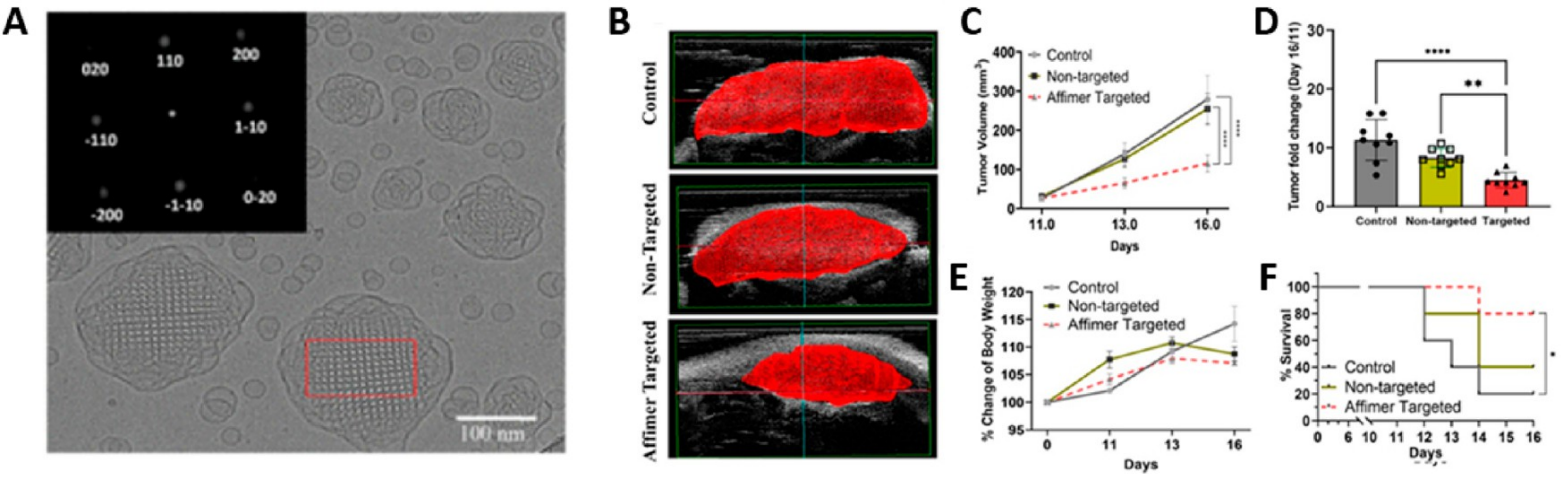
(A) Cryo-TEM image of cubosome showing its structure and morphology. (B–D) Efficacy of tumor growth reduction in the Affimer targeted delivery, nontargeted delivery and control group. (E, F) Body weight change and survivability of the three groups of mice showing significantly high therapeutic efficacy in the Affimer targeted group. Adopted from ref (13). Available under a CC-BY 4.0 license.

Cisplatin is commonly used for the treatment of colorectal cancer (CRC) but it is also linked with severe side effects and the development of drug resistance. In a study done by Umar et al., nanocubosomes were synthesized with encapsulated cisplatin and a cisplatin-metformin mixture for testing on CRC cells HCT-116.^160^ Comparing nanocubosomal formulation to free cisplatin, the former showed a more potent cytotoxic impact.^161,162^ The addition of metformin, a type of indirect mTOR inhibitor, to cisplatin nanocubosomes, significantly increased the cytotoxic impact. Through the blocking of many metabolic processes, specifically Akt/mTOR, and AMPK/mTOR, the CRC cell death was triggered as shown in the study. Following nanocubosomal therapy, p-Akt (Ser473) levels were also suppressed, further inhibiting mTOR. Additionally, drug-loaded nanocubosomes caused a significant rise in ROS levels, which was shown by a rise in NADPH oxidase, a reduction of LDH, and an accompanying rise in caspase-3. In a recent study, our group demonstrated that cubosomes could be successfully targeted to colorectal cancer cells *in vivo* using a carcinoembryonic antigen binding protein known as “Affimer”. Post targeted delivery, the drug release showed significant tumor inhibition and improved the overall survivability of mice without showing any toxicity (Figure 7).^13^

#### 6.5.5 Liver Cancer Treatment

Nasr et al. formulated 5-Fluorouracil (5-FU) loaded cubosome and studied its therapeutic impact in liver cancers both *in vitro* and in a rat model.^147^ The release of 5-FU from cubosome was almost 50% slower compared to free 5-FU as studied from in vitro analysis. The cubosomal composition considerably boosted 5-FU liver uptake five times higher than free 5-FU according to in vivo biodistribution tests. Rats administrated with cubosome formulation had more hepatocellular damage, according to histological and serum serological data. These findings show that cubosome nanoparticles carrying 5-FU for liver cancer delivery were successfully developed.^147^

#### 6.5.6 Ovarian Cancer Treatment

Icariin (ICA) has the ability to inhibit cancer cell proliferation, yet it has minimal clinical applicability due to its poor solubility in water. In comparison to free ICA, ICA-loaded cubosomes formulation (ICA-Cubs) showed increased cytotoxicity and apoptosis capability when used against ovarian cancer cell lines Caov-3 and SKOV-3 as studied by Varghese et al.^11^ The noncancerous EA.hy926 endothelial cells exposed to the ICA-cubosome had a comparatively minimal cytotoxic response. Its increased effectiveness in comparison to the free ICA may be attributable to ICA’s increased cellular permeability and bioavailability. In the SKOV-3 cell line, ICA-cubosome induced overexpression of caspase-3 and p53 along with reactive oxygen species (ROS). In a nutshell, the cubosome mediated delivery of ICA may offer a potential route to an effective ovarian cancer treatment.^163^

#### 6.5.7 Cervical Carcinoma

Victorelli et al. developed cubosomes using the cationic lipid DOTAP, to enhance mucoadhesion and enable the topical delivery of lipophilic medications like curcumin to the vagina.^152^ It was observed in their study that vaginal epithelium maintained the curcumin released from the cubosomes indicating that the technique has a possibility for topical delivery. Further in their study, it was observed that Hela cells were capable of internalizing the cubosomes, and these nanoparticles improved the anticervical cancer effects of curcumin, according to cellular uptake and in vitro cytotoxicity studies.^152^ The curcumin-loaded cubosomes had lowered the antiangiogenic effect of blood vessels after 4 h of treatment, as observed in the *in vivo* investigation utilizing the CAM model. These encouraging findings indicate that cubosomes represent a very viable foundation for the inclusion of lipophilic medications for the topical therapy of cervical cancer and other diseases.

#### 6.5.8 Breast Cancer Therapy

While hormone therapy and chemotherapy are sometimes combined in the course of treatment for breast cancer, the effectiveness of this approach is constrained by the different pharmacokinetic properties of the two drugs, which prevent their simultaneous and targeted delivery to cancer cells.^164^ Mokhtar et al. developed a hybrid carrier system using cubosome formulation for the simultaneous targeted administration of methotrexate (MTX) and the aromatase inhibitor exemestane (EXE).^164^ Methotrexate (MTX) and exemestane (EXE) were codelivered using cubosome nanoparticles that were lactoferrin-targeted. While MTX was chemically attached to lactoferrin through the carbodiimide process, EXE was physically loaded into the cubosomes.^164^ This demonstrated that targeted dual drug-loaded cubosome might be a feasible alternative for combination hormonal chemotherapy, allowing for additional *in vivo* research to demonstrate their effectiveness in a preclinical breast cancer model.

## 7 Potential Challenges in Translating Lipidic Nanocarriers As Therapeutic Agents in Precision Medicine to Clinical Settings

**Figure 8:**
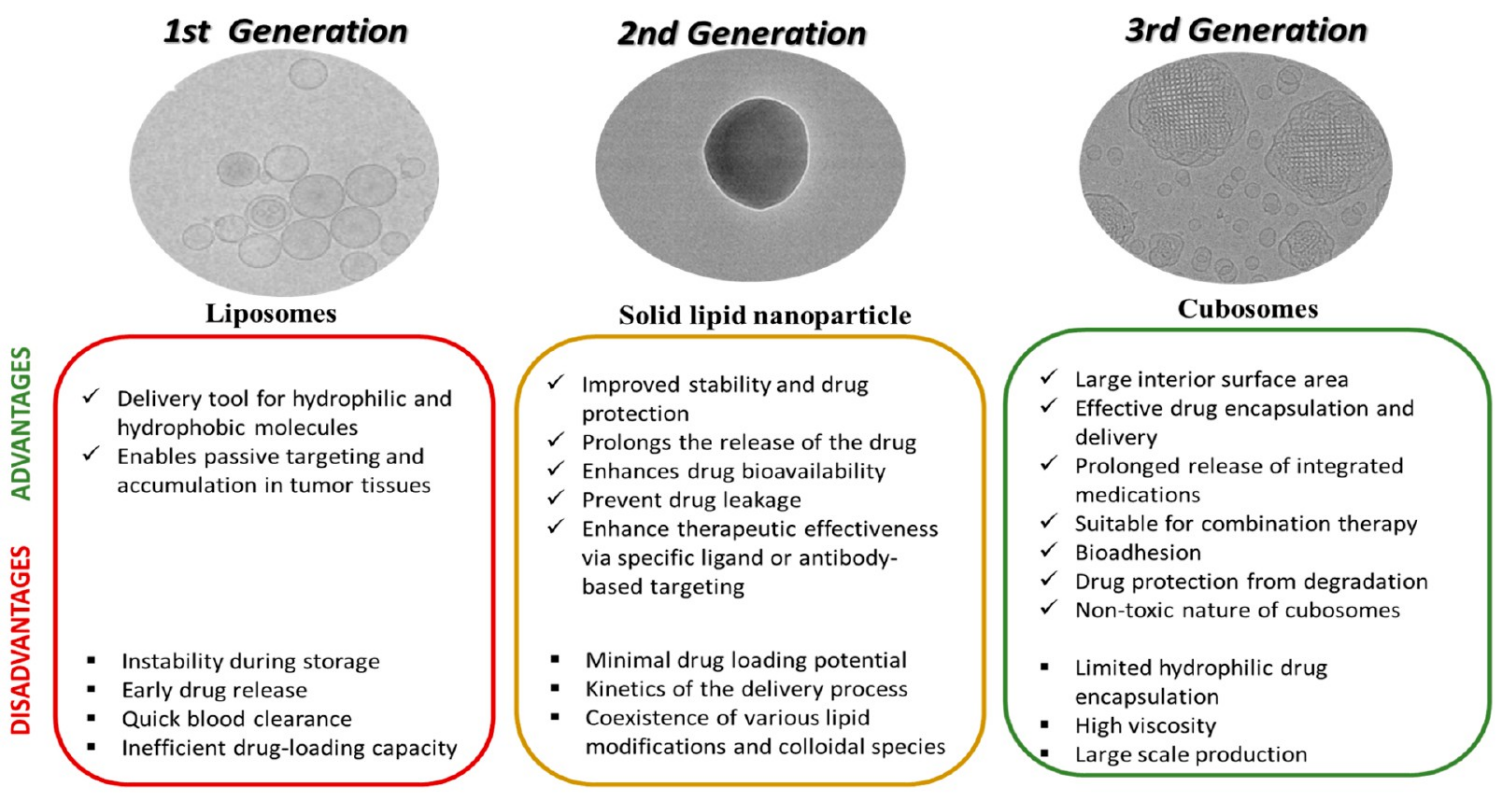
Summary of the three generations of lipidic nanocarriers with their advantages and drawbacks. Figures have been reproduced with permission from ref (165), (166), and (13). Copyright 2014 Elsevier, 2018 Elsevier, and 2022 American Chemical Society.

Although vast research findings showed the promising potential of lipidic nanocarriers as therapeutic agents in precision medicine, there are several key challenges that might impede the translation into clinical application (Figure 8). Comprehensive preclinical and clinical trials are required to evaluate the safety, toxicity, or adverse effects of the lipidic nanocarriers as therapeutic agents. More research is needed incorporating specific ligands or receptors onto the nanocarriers to ensure successful targeted delivery on the specific cells or tissues. Additionally, heterogeneity in patients requires tailoring treatments to individual patients based on their genetic, molecular, and clinical characteristics. Another common challenge of therapeutic agents is to bypass biological barriers, such as the blood-brain barrier. Hence, developing adaptable lipidic nanocarrier platforms that can be customized for each patient and can cross the blood-brain barrier is imperative. With regard to commercialization, maintaining the quality and reproducibility of mass production of lipidic nanocarriers is crucial and can be difficult. Ideally, nanomaterial-based therapies should be low-cost and can be available to the mass population. However, developing nanomaterial-based therapies can be expensive. Another stumbling block is getting the regulatory approval for nanomaterial-based therapies can be complex and tedious.

## 8 Conclusion

In the past decade, liposomal formulations including solid lipid nanoparticles have emerged as a promising avenue in the field of drug delivery, offering numerous advantages such as enhanced drug solubility, improved bioavailability, and targeted delivery to specific tissues or cells. Over the years, liposomal formulations have proven effective in delivering a wide range of therapeutic agents, from conventional chemotherapeutic drugs to newer biologics and nucleic acid-based therapies. They have played a crucial role in mitigating the limitations associated with traditional drug delivery methods, such as poor drug stability, off-target effects, and limited therapeutic index. However, these traditional liposomes and solid lipid nanoparticles have a number of disadvantages and limitations. In this respect, the new generation of cubosome nanocarriers has addressed those issues such as higher drug loading capacity, enhanced stability, sustained release, improved tissue penetration, versatile shape and size control, reduced immunogenicity, biocompatibility, and ease of functionalization as a step forward in this field of research. Cubosomes have been intensively researched as a therapeutic delivery vehicle in a variety of disorders, including cancer. Despite the fact that various milestones remain to be reached, research data and preclinical trial results point to cubosomes as a promising next-generation liposomal formulation for furthering the treatment of a variety of infections and disorders, including a wide range of cancers.
